# Supplementary material for: A multicellular way of life for a multipartite virus
Source: eLife. 2019 Mar 12;8:e43599. doi: 10.7554/eLife.43599 (PMC6414197; doi:10.7554/eLife.43599)
Supplement: Supplementary file 1. [file elife-43599-supp1.docx]

**Supplementary file 1: Tables**

**Table S1. Segments (FISH) and M-Rep protein (immuno-fluorescence) detection in infected plants**

^a^ Code number of individual plants and petioles harvested on these plants

^b^ Time of infection at which the petiole has been harvested, expressed in days post infection

^c^ Segments pair or segment/M-Rep protein detection, identity of x and y is indicated

^d^ Total number of cells in the petiole where at least one of the two analyzed x and y was detected

^e^ Number of cells in the petiole where only x was detected

^f^ Number of cells in the petiole where only y was detected

^g^ Number of cells in the petiole where both x and y were detected

^h^ Ratio of the respective segment’s copy number within a segment pair estimated by qPCR

nt: not tested**Table S2. Oligonucleotides used to prepare segment-specific fluorescent probes**

^a^ Name of the primers or oligonucleotide probe

^b^ Sequence of the primer or of the oligonucleotide probe

^c^ Size of the target sequence corresponding to the size of amplicons or oligonucleotide probes

^d^ Identity of the FBNSV genome segment targeted by the probe

^e^ The oligonucleotide probes are described in the Methods section

**Table S3. Confocal microscope settings for acquisition of images shown in the figures**

^a^ Numbers and letters correspond to figures and images, respectively

^b^ Single optical section (Plane: P) or a successive series of optical sections (stack: S)

^c^ Number of successive optical sections in the stack

^d^ Thickness of each optical section

^e^ Resolution of the original images, number of division per image is similar for width and length

^f^ Number of bit encoded at image acquisition

^g^ Optical magnification associated to distinct objectives

^h^ Digital zoom magnification

^i^ The acquisition time per pixel is given in μs

^j^ Laser strength is given in % of its maximum capacity

^k^ Photomultiplier gain is given in Volts
