# Supplementary material for: A multicellular way of life for a multipartite virus
Source: eLife. 2019 Mar 12;8:e43599. doi: 10.7554/eLife.43599 (PMC6414197; doi:10.7554/eLife.43599)
Supplement: Transparent reporting form [file elife-43599-transrepform.docx]

***eLife’s* transparent reporting form**

We encourage authors to provide detailed information *within their submission* to facilitate the interpretation and replication of experiments. Authors can upload supporting documentation to indicate the use of appropriate reporting guidelines for health-related research (see [EQUATOR Network](http://www.equator-network.org/%20)), life science research (see the [BioSharing Information Resource](https://biosharing.org/)), or the [ARRIVE guidelines](http://www.plosbiology.org/article/info:doi/10.1371/journal.pbio.1000412) for reporting work involving animal research. Where applicable, authors should refer to any relevant reporting standards documents in this form.

If you have any questions, please consult our Journal Policies and/or contact us: [editorial@elifesciences.org](mailto:editorial@elifesciences.org).

**Sample-size estimation**

- You should state whether an appropriate sample size was computed when the study was being designed
- You should state the statistical method of sample size computation and any required assumptions
- If no explicit power analysis was used, you should describe how you decided what sample (replicate) size (number) to use

Please outline where this information can be found within the submission (e.g., sections or figure legends), or explain why this information doesn’t apply to your submission:

Sample size:

The number of individual cells quantified for the co-localisation of segments/protein or for the segment accumulation correlation tests was a compromise between the required statistical power and evident practical constraints inherent to our biological system. The virus being phloem-restricted, the number of cells containing viral material that is detected within a series of cross section within an infected petiole is very low and impossible to predict. We thus decided to analyze a large number on plants infected independently. All cells containing viral material in cross sections from each of these plants were imaged. A total of 49 independent plants and 56 petioles have been analyzed. The information on sample size (number of observed cells per infected plant) can be found in the text article and in Table 1, Table S1, Figure S4 and Table S4.

Randomization and Blinding of the experiments:

Randomization is not relevant for our study. We simply infect plants with a multipartite virus and monitor the accumulation of distinct viral genome segments in individual cells using a combination of confocal microscopy and qPCR. Each infected plant reflects what is going on during the course of one intra-host productive infection.

Blinding is not relevant in our study. The investigator localizes, quantifies and compares two labeled segments (either green or red label) in each experiment. Knowing (or not) which segment is green and which one is red cannot change their respective localization or their uncorrelated accumulation intensity.

**Replicates**

- You should report how often each experiment was performed
- You should include a definition of biological versus technical replication
- The data obtained should be provided and sufficient information should be provided to indicate the number of independent biological and/or technical replicates
- If you encountered any outliers, you should describe how these were handled
- Criteria for exclusion/inclusion of data should be clearly stated
- High-throughput sequence data should be uploaded before submission, with a private link for reviewers provided (these are available from both GEO and ArrayExpress)

Please outline where this information can be found within the submission (e.g., sections or figure legends), or explain why this information doesn’t apply to your submission:

That the accumulation of a given segment is independent from that of another has been consistently verified in 10 plants infected independently (as well as the fact that the segments rarely co-occur in the same individual cells) for the segment pair R/S (encoding replication/capsid proteins). This was further verified for the pair of segments R/M (replication/movement; two plants infected independently) and for the pair of segments S/M (capsid protein/movement; three plants infected independently).

That the protein M-rep (expression product of segment R) is most often present in the cells where another segment replicates has been evaluated on ten plants infected independently for segment S and four plants infected independently for segment R.

None of these repeats yielded inconsistent results: i) one segment of a pair rarely co-occured with the other in all repeats (Table S1, Table S4), ii) the accumulation of any segment of a pair was never correlated to the accumulation of the other for all pairs considered (Table 1, Table S4), iii) the protein M-Rep is present in most cells where any segment replicates in all plant/petiole repeats (Figure 3C and Table S4).

Data exclusion:

Because the segment-accumulation correlation tests were performed in each individual petiole, all petioles where less than 10 cells could be analyzed have been discarded from the analysis (individual petioles used in our data set yielded between 15 and 67 positive cells, all data in Tables 1, S1 and S4). Indeed we considered a priori that the absence of correlation between the accumulation of two distinct segments could be due in these cases to a too low n and this data exclusion can thus only be conservative for our conclusion.

**Statistical reporting**

- Statistical analysis methods should be described and justified
- Raw data should be presented in figures whenever informative to do so (typically when N per group is less than 10)
- For each experiment, you should identify the statistical tests used, exact values of N, definitions of center, methods of multiple test correction, and dispersion and precision measures (e.g., mean, median, SD, SEM, confidence intervals; and, for the major substantive results, a measure of effect size (e.g., Pearson's r, Cohen's d)
- Report exact p-values wherever possible alongside the summary statistics and 95% confidence intervals. These should be reported for all key questions and not only when the p-value is less than 0.05.

Please outline where this information can be found within the submission (e.g., sections or figure legends), or explain why this information doesn’t apply to your submission:

The nature of the statistical tests is indicated in the text and in the legends of Figures and tables.

For correlation tests, the n, r, F and p values are provided in Table 1, Figure S2 and Figure S3

(For large datasets, or papers with a very large number of statistical tests, you may upload a single table file with tests, Ns, etc., with reference to sections in the manuscript.)

**Group allocation**

- Indicate how samples were allocated into experimental groups (in the case of clinical studies, please specify allocation to treatment method); if randomization was used, please also state if restricted randomization was applied
- Indicate if masking was used during group allocation, data collection and/or data analysis

Please outline where this information can be found within the submission (e.g., sections or figure legends), or explain why this information doesn’t apply to your submission:

N/A: All regression analyses have been performed per individual infected plant and thus group allocation is not an issue.

In Figure S3, some experimental repeats were pooled and analyzed with a GLM model. Pooled data are those corresponding to similar experiments and so, again, there is no group allocation issues.

**Additional data files (“source data”)**

- We encourage you to upload relevant additional data files, such as numerical data that are represented as a graph in a figure, or as a summary table
- Where provided, these should be in the most useful format, and they can be uploaded as “Source data” files linked to a main figure or table
- Include model definition files including the full list of parameters used
- Include code used for data analysis (e.g., R, MatLab)
- Avoid stating that data files are “available upon request”

Please indicate the figures or tables for which source data files have been provided:

All data are available in the manuscript and in Supplemental Information.

Raw data of all quantified green and red fluorescence within individual cells of infected plants are provided as a separate EXCEL supplementary file: Table S4.

To allow repeat/reproduce all correlation tests, the 508 raw/unprocessed images (.lsm format) used for preparing all figures and for fluorescence quantification in individual cells have been deposited in the public repository figshare. They can be accessed at the DOI: [10.6084/m9.figshare.5981968](https://doi.org/10.6084/m9.figshare.5981968)
